# Congenital duodenal obstruction with situs inversus totalis: Report of a rare association and discussion

**DOI:** 10.4103/0971-9261.43029

**Published:** 2008

**Authors:** Satendra Sharma, Kumar Abdul Rashid, Ravi Dube, G. K. Malik, R. K. Tandon

**Affiliations:** Department of Pediatric Surgery, King George's Medical University, Lucknow, India

**Keywords:** Babygram, congenital duodenal obstructions, reverse double bubble, situs inversus

## Abstract

This report is to present and discuss an extremely rare association of situs inversus with duodenal atresia in an 11-day-old male neonate born full term and weighing 1.9 kg. The baby presented with recurrent bilious vomiting. Babygram revealed situs inversus and duodenal obstruction. Echocardiography showed dextrocardia with a small ASD. Exploration confirmed a duodenal diaphragm with a central perforation between the third and fourth part of the duodenum and situs inversus. The literature search revealed 20 cases reported so far.

## INTRODUCTION

Situs inversus is a rare condition causing mirror image positioning of abdominal and thoracic viscera with an estimated frequency of about one in 10000 of normal population.[1,2] It is commonly associated with other serious cardiac and splenic malformations.[3,4] Duodenal atresia is estimated to appear with an incidence of about one in 4000 to one in 15000 live births and about half of them have an associated congenital anomaly of another organ system.[4,5] The association of congenital duodenal obstruction and situs inversus is extremely rare, with only 20 cases reported so far in the literature.[4]

## CASE HISTORY

**Figure 1:**
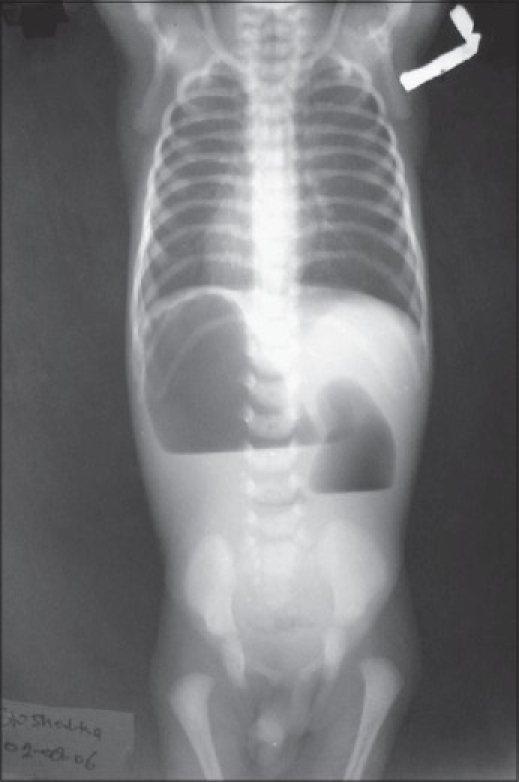
Babygram showing dextrocardia and a reverse double bubble

An 11-day-old full-term male baby weighing 1.9 kg presented with recurrent bile-stained vomiting after feedings. On examination, his abdomen was scaphoid with mild upper abdominal distension. An orogastric tube was inserted that persistently drained bile. The babygram revealed dextrocardia with a reverse double bubble sign and no distal gas [Figure 1]. An echocardiogram showed dextrocardia with a 3 mm ASD. The neonate underwent laparotomy on the 12^th^ day of life. The stomach and spleen were found on the right side while the liver was found on the left side. The cecum and appendix were located in left lumbar region lying on the left side. Ladd's bands were found with narrowed base of mesentery. The Ladd's bands were divided and whole duodenum was mobilized. It was found hypertrophied and dilated up to the beginning of fourth part beyond which bowel was unused and narrow. Duodenotomy was done at this site. A duodenal diaphragm with a central pin-hole perforation was found. This was excised and duodenotomy was closed. Appendicectomy was not done. Patient had an uneventful recovery and was discharged in satisfactory condition.

## DISCUSSION

Congenital duodenal obstruction is an uncommon condition; with an estimated incidence of about one in 4000 to one in 15000 live births and about half of them having an associated congenital anomaly of another organ system.[3,4] Down syndrome is the most common association found in about 30% of cases and congenital heart disease in 4-49% of cases.[3,5] Embryologically, failure of recanalization, after its stage of obliteration by rapidly growing epithelium, or arrest in duodenal growth at any stage results in the development of duodenal atresia, stenosis or diaphragm with or without an aperture.[3–5] Situs inversus is a defect in the normal development of the human left-right asymmetry during embryogenesis that results in laterality defects with cardiovascular, abdominal, and pulmonary malformations.[2,4] Etiologic factors in situs inversus are unknown; familial occurrence suggests multiple inheritance patterns.[2] Mirror-image dextrocardia is a common form of cardiac malposition and is almost always associated with situs inversus of the abdominal organs, called *situs inversus totalis*.[6] The prevalence of situs inversus totalis seems to range between one in 8000 and one in 25,000.[2,4,6] It is a condition in which the morphologic right atrium is on the left and the morphologic left atrium is on the right. The normal pulmonary anatomy is reversed such that the left lung has three lobes and the right lung has two. In addition, the liver and gallbladder are located on the left, and the spleen and stomach are on the right. The remaining internal structures also mirror the normal situation. The association of congenital duodenal obstruction and situs inversus is extremely rare, annular pancreas being the most common, reported in six patients, followed by duodenal web in four patients.[4,7] Among the various causes of duodenal obstruction reported in association with situs inversus, pre-duodenal portal vein, duodenal stenosis and complete duodenal atresia have also been reported.[4] Situs inversus can be asymptomatic, found incidentally during laparotomy or autopsy. But, when associated with congenital duodenal obstruction, it can present early in the neonate. The diagnosis of congenital duodenal obstruction in association with situs inversus is made easily on a plain babygram when a reverse double bubble sign is seen with reversed heart shadow. Confirmation of the diagnosis can be done by a contrast meal and follow through especially in those with partial duodenal obstruction secondary to a duodenal web with a central aperture. Echocardiography to document the presence or absence of associated congenital cardiac anomalies is an important preoperative investigation. The treatment of congenital duodenal obstruction with or without associated situs inversus is the same. If the obstruction is caused by an intra-luminal web, as in our case, excision and duodenoplasty is the treatment of choice. Duodenoduodenostomy is preferred by some to avoid iatrogenic biliopancreatic injury because of proximity of ampulla of Vater to site of the duodenal web.[5,7–9]

## References

[1] Le Wald LT (1925). Complete transposition of the viscera: A report of twenty-nine cases, with remark on etiology. JAMA.

[2] Casey B (2001). Genetics of human situs abnormalities. Am J Med Genet.

[3] Bailey PV, Tracy TF, Connors RH, Monney DP, Lewis JE, Weber TR (1993). Congenital duodenal obstruction: A 32 years review. J Pediatr Sur.

[4] Nawaz A, Matta H, Hamchou M, Jacobez A, Trad O, Al Salem AH (2005). Situs inversus abdominus in association with congenital duodenal obstruction: A report of two cases and review of the literature. Pediatr Surg Int.

[5] Akhtar J, Guiney EJ (1992). Congenital duodenal obstruction. Br J Surg.

[6] Gutgesell HP, Garson A, Bricker JT, Fisher DJ, Neish SR (1997). Cardiac malposition and heterotaxy. The Science and Practice of Pediatric Cardiology.

[7] Adeyemi SD (1988). Combination of annular pancreas and partial situs inversus: A multiple organ malrotation syndrome associated with duodenal obstruction. J Pediatr Surg.

[8] Luchtman M, Golan Y, Heldenberg D, Kessler F (1933). Situs inversus abdominus in association with duodenal obstruction and internal hernia. Am J Perinatol.

[9] Akel S, Halabi J, Shawis R (1998). Abdominal situs inversus with congenital duodenal stenosis: Rare association. Eur J Pediatr Surg.

